# One Health: Past Successes and Future Challenges in Three African Contexts

**DOI:** 10.1371/journal.pntd.0002884

**Published:** 2014-05-22

**Authors:** Anna L. Okello, Kevin Bardosh, James Smith, Susan C. Welburn

**Affiliations:** 1 Division of Pathway Medicine and Centre for Infectious Diseases, School of Biomedical Sciences, College of Medicine and Veterinary Medicine, The University of Edinburgh, Edinburgh, United Kingdom; 2 Centre of African Studies, School of Social and Political Science, The University of Edinburgh, Edinburgh, United Kingdom; 3 Department of Geography, Environmental Management and Energy Studies, University of Johannesburg, Johannesburg, South Africa; Swiss Tropical and Public Health Institute, Switzerland

## Abstract

**Background:**

The recent emergence of zoonotic diseases such as Highly Pathogenic Avian Influenza (HPAI) and Severe Acute Respiratory Syndrome (SARS) have contributed to dominant Global Health narratives around health securitisation and pandemic preparedness, calling for greater co-operation between the health, veterinary and environmental sectors in the ever-evolving One Health movement. A decade later, One Health advocates face increasing pressure to translate the approach from theory into action.

**Methodology/Principal Findings:**

A qualitative case study methodology was used to examine the emerging relationships between international One Health dialogue and its practical implementation in the African health policy context. A series of Key Informant Interviews (n = 32) with policy makers, government officials and academics in Nigeria, Tanzania and Uganda are presented as three separate case studies. Each case examines a significant aspect of One Health operationalisation, framed around the control of both emerging and Neglected Zoonotic Diseases including HPAI, Human African Trypanosomiasis and rabies. The research found that while there is general enthusiasm and a strong affirmative argument for adoption of One Health approaches in Africa, identifying alternative contexts away from a narrow focus on pandemics will help broaden its appeal, particularly for national or regionally significant endemic and neglected diseases not usually addressed under a “global” remit.

**Conclusions/Significance:**

There is no ‘one size fits all’ approach to achieving the intersectoral collaboration, significant resource mobilisation and political co-operation required to realise a One Health approach. Individual country requirements cannot be underestimated, dismissed or prescribed in a top down manner. This article contributes to the growing discussion regarding not whether One Health should be operationalised, but *how* this may be achieved.

## Author Summary

The One Health movement requires more robust evidence around its practical implementation if it is to truly become a way forwards for addressing health issues at the human, animal and ecosystem interface. The research in this paper discusses some of the recent successes and challenges with both Emerging and Neglected zoonoses in the sub-Saharan Africa context. Through speaking to various human and animal health practitioners and policy makers in Uganda, Nigeria and Tanzania, the authors have created three case studies highlighting the various successes of the approach to date, but also clarifying areas where the approach will take longer to implement, often as a result of the wide institutional and policy changes required in many countries. The authors conclude that whilst the “goodwill” is certainly there, the reality of planning, executing and budgeting for joint interventions – particularly at the national or regional level – proves in many cases more difficult than first thought. It is hoped however that through gaining better insight from those charged with the decision-making in these countries, One Health practitioners will be encouraged to build on the momentum through addressing some of the issues that arise with its implementation.

## Introduction

**Figure 1:**
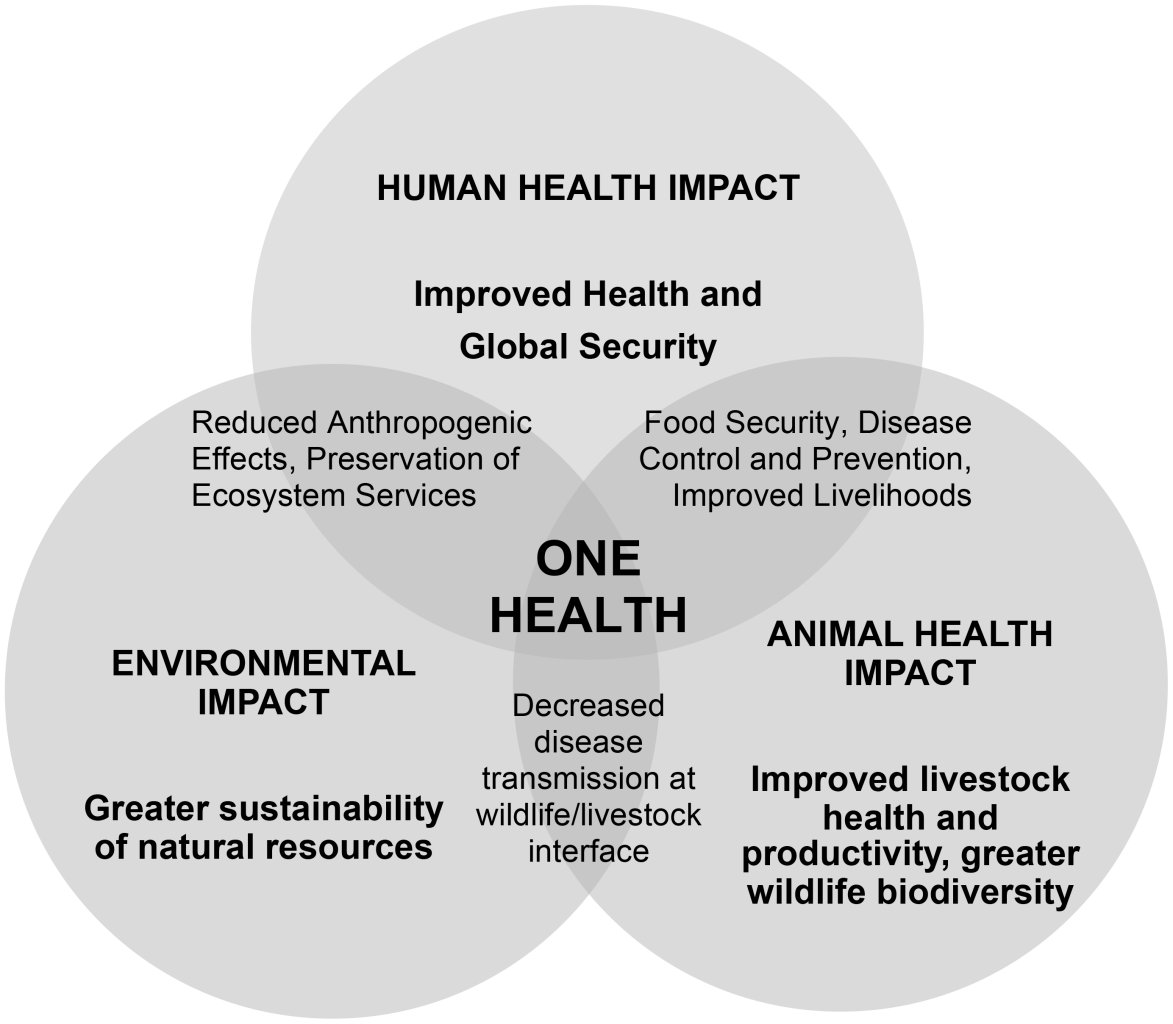
Thematic representation of One Health depicting potential added benefits of sectoral overlap (grey areas).

One Health acknowledges the close relationships between humans, animals and ecosystems, promoting the potential added benefits to each sector or species that emerge as a result of its operationalisation (Figure 1). Whilst attempts to categorically define One Health are many and varied, the general consensus that it promotes a transdisciplinary, collaborative “whole of society” approach towards global health in the 21^st^ century remains key [1]–[4].

The unprecedented financial and political response to H5N1 avian influenza at the turn of the 21^st^ century facilitated the development of global intersectoral alliances, creating a unique policy space for agencies, governments and institutes to collaborate under a large scale One Health banner for the first time [3],[4]. There is currently a strong drive to maintain the momentum and alliances established during the Global Response to Avian Influenza (GRAI), with advocates promoting One Health as an approach towards various other aspects of international and regional health governance. However in the absence of a specific disease threat, examples of national commitment to One Health are increasingly difficult to find, particularly in developing countries. The argument for inter-ministerial platforms to co-ordinate policy and action for zoonoses control is well founded. However whilst One Health is theoretically - and arguably economically - attractive, significant political will and state capacity is required to overcome existing institutional and financial barriers to its implementation; particularly in developing countries where numerous health and development priorities compete for attention and programmatic funding. Identification and critical analysis of current examples is required if One Health is to be perceived as anything other than an “attempt to grab funds on the tail-end of the avian influenza bonanza” [4].

Africa is a relevant continent for the examination of One Health policy, particularly for the control of endemic and “neglected” zoonotic diseases [5]. Although Asia has been the recent focus regarding high profile emerging zoonotic disease outbreaks, Africa has historically been home to some of the most striking examples of disease spill over from animals including HIV, Rift Valley Fever and Ebola. Additionally, it is estimated one third of Africa's agricultural Gross Domestic Profit is obtained through livestock production [6]. Whilst significant economic gains could be realised on the continent through control of production-limiting zoonoses including the trypanosomiases, brucellosis, cysticercosis and anthrax, the existing socioeconomic evidence available to promote concrete policy shifts towards multisectoral approaches is currently lacking. In addition to the socioeconomic evidence, documenting the successes and challenges of existing One Health platforms, as experienced by those driving disease control policies on the continent, is also urgently required. Through interviewing a selection of respondents currently at the forefront of policy development for zoonoses control in Uganda, Nigeria and Tanzania, attempts have been made to address this latter issue.

## Methods

**Figure 2:**
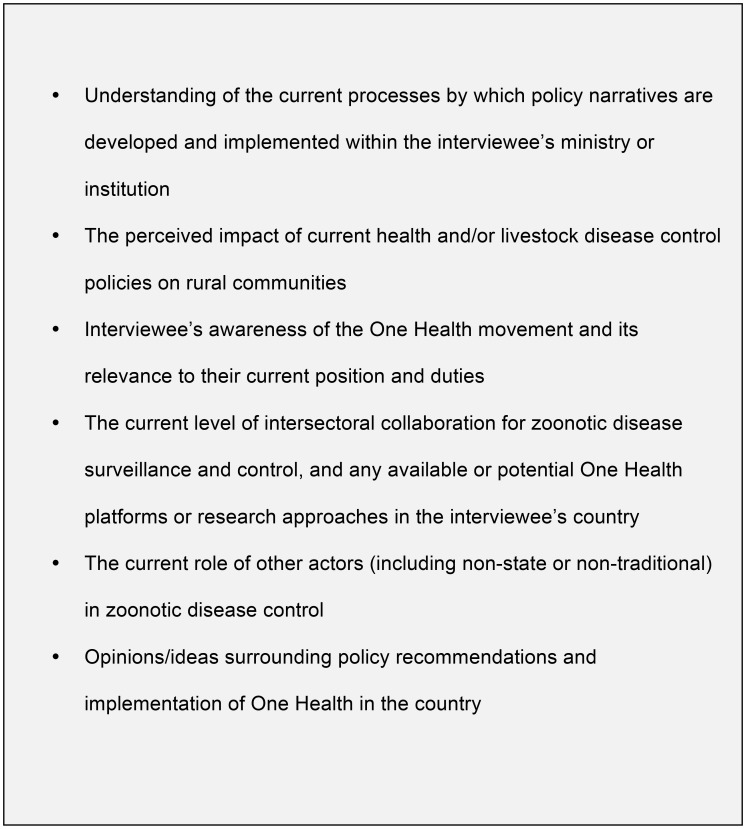
Semi-structured interview theme guide.

A preliminary review identified that despite the ubiquitous international promotion of One Health through various meetings, agreements, frameworks and pledges, relatively few examples of successful long term adoption of the approach existed, particularly in sub-Saharan Africa [7]. A qualitative case study methodology [8] was applied in three African contexts in order to understand how – or where – a One Health policy approach may be appropriate to the control of diseases of regional or national importance. A total of 32 Key Informant Interviews were held with key policy actors in Uganda, Nigeria and Tanzania, including officials from the Ministries of Health and Agriculture, academia and international research institutes (Table 1). These countries were chosen given they have reported higher than average burdens of endemic zoonotic disease [5], and were key International Cooperation Partner Countries (ICPCs) of the European Commission's Integrated Control of Neglected Zoonoses (ICONZ) project through which the research was undertaken [9]. Individual interview respondents were selected using a snowball sampling technique; a type of purposive sampling whereby existing local networks direct the researcher to further potential participants [8]. Given the relatively “closed” doors and time constraints common to government officials across many African ministries, snowball sampling was deemed the most sensible - and in many cases the only available - technique to ensure that interviews were secured from a wide variety of sectors and ministerial levels. With the exception of one international researcher, interview respondents were all nationals of the three focus countries, representing several sectors and governance levels as outlined in Table 1. Whilst the semi-structured interview approach allowed for a certain degree of flexibility to reflect respondents' areas of expertise and experience, several key themes exploring intersectoral collaboration in the context of disease control were used as a general interview guide (Figure 2). Verbal consent was obtained prior to the commencement of all interviews, which were then documented via handwritten notes and voice recordings if the respondent agreed. Resulting transcripts were then manually coded according to the various emerging themes and topics, from which several context-specific narratives were then developed. The resulting observations and recommendations, discussed in the remainder of this article, outlines the various personal experiences of those in the “driving seat” of disease control in the three countries, potentially increasing the understanding of how One Health's application can extend to a wider variety of diseases and national contexts outside the GRAI.

**Table 1:** Interview respondent distribution by domain of expertise.

Interviewee Domain	Number of Respondents
Policy – Central or Federal government (Animal Health Sector)	6
Policy – Central or Federal government (Human Health Sector)	4
Policy – State, District or local government (Animal Health Sector)	6
Policy – State, District or local government (Human health sector)	3
Academic or research institute (national)	3
International representative	1
Health practitioner (Animal health sector)	6
Health practitioner (Human health sector)	3
**TOTAL**	**32**

## Results/Discussion

### Case Study One: Twenty Years of One Health - The Co-ordinating Office for the Control of Trypanosomiasis in Uganda (COCTU)

Human African Trypanosomiasis (HAT), or “Sleeping Sickness”, is a Neglected Tropical Disease of significant public health importance across much of Africa, with Nagana the corresponding disease in livestock. Transmitted by the *Glossina* species of tsetse fly, trypanosomiasis manifests in humans as either an acute or chronic form caused by *T. br. rhodesiense* and *T. br. gambiense* respectively. Presently the only country with foci of both forms of this fatal human disease, Uganda has suffered from devastating epidemics and outbreaks since the beginning of the 20^th^ Century. To date the two forms have been confined to separate geographical foci in Uganda, facilitating surveillance and treatment. More recently however, country-wide movements of infected cattle - an essential reservoir of the *T. b. rhodesiense* parasites responsible for acute human disease in Uganda - have fuelled fears of disease convergence [10],[11]. The intersectoral approach required for HAT control “lies at the heart of African rural development” [12], providing a relevant case study through which to examine One Health.

#### One health “by accident”

Formed by a parliamentary Act (Statute 16) on the 8^th^ of October 1992, the Co-ordinating Office for the Control of Trypanosomiasis in Uganda (COCTU) is the secretariat of a permanently funded interministerial platform, mandated to co-ordinate policy and oversee all Human and African Trypanosomiasis control in the country [13]. Seated within Uganda's Ministry of Agriculture, Animal Industries and Fisheries (MAAIF) and endorsed by the Office of the Prime Minister for the better part of two decades, COCTU is a unique example of Uganda's commitment to One Health long before the approach became “fashionable”.

The basis for COCTU's foundation lay in a major *T. b rhodesiense* epidemic in the late 1980s, where significant human and financial resource inputs necessitated a change from the disaggregated “silo” approach of past control programmes in order to more effectively deal with the human disease burden: *“Prior to (COCTU's formation) there existed very many players; no-one knew what was happening in the other sphere. The 1988 epidemic rapidly brought down cases by 1990 because vets, medics, vector control and researchers were all in the same area using known amounts of money. It was very controlled and co-ordinated” (Key Informant 1, Uganda)*. Those involved in the joint intervention of the early 1990's depicted COCTU as a “*good arrangement*” *(Key Informant 2, Uganda)* to sustainably promote the added sectoral benefits arising from a One Health approach towards HAT control in the country.

#### The challenges of establishing and maintaining a permanent inter-ministerial platform

The institutional vision required to initiate and subsequently sustain the COCTU secretariat should not be underestimated, nor is it without its challenges. Under Ugandan law, any permanent platform must be housed within a single ministry; respondents felt the decision to house COCTU in MAAIF resulted from the major drive for trypanosomiasis control by the agricultural sector at the time. However ministerial ownership, particularly regarding long term financial support of the initiative, was cited as an ongoing challenge to the secretariat. Some felt partners under-budgeted for their various components, assuming MAAIF would cover the deficit. Conversely, as the MAAIF budget allows only for administrative activities undertaken by the secretariat, control interventions in the animal reservoir still require funding from a separate budget line, leading to accusations that MAAIF is “*taking advantage*” of the structural weaknesses in COCTU *(Key Informant 3, Uganda)*. The importance for roles and responsibilities to be agreed and understood by all stakeholders involved in One Health approaches cannot be underestimated, particularly regarding financial resource allocation. When asked whether the COCTU Secretariat would be better suited to ownership under another ministry, a (non-MAAIF) respondent replied: *“Wherever it is housed, it must be well managed…even if it sits in the Ministry of Health it will have the same problems with day to day running” (Key Informant 4, Uganda)*.

Despite ongoing financial challenges, the Ugandan ownership and high-level political endorsement of COCTU demonstrates how One Health success is likely much more sustainable when owned and paid for nationally, not driven by external funding as the majority of One Health activities have been to date; *“There are many more problems that worry people in Uganda than avian influenza. If we wanted to kick off One Health here, you promote it as something to benefit people” (Key Informant 5, Uganda)*. Ultimately, high-level political backing was deemed a key element for One Health success; *“The first thing is to make (One Health) appreciated by the leadership of a country; if they accept it (then) you have recognised the problem” (Key Informant 6, Uganda)*.

### Case Two: After the Crisis - Maintaining One Health Momentum in Post-H5N1 Nigeria

On the 6^th^ of February 2006, Nigeria reported Africa's first case of H5N1 Highly Pathogenic Avian Influenza in a commercial poultry farm in Kaduna state. The political and financial backing for control was unprecedented - *“the government was giving money before they were even asked to” (Key Informant 1, Nigeria)* - with an alleged USD $50 million credit received from the World Bank to commence activities [14],[Key Informant 1 Nigeria]. Pressure from external agencies resulted in the “Nigeria Avian Influenza Emergency Control Preparedness and Response Project”; a three year action plan that promoted the added benefits of a One Health approach through its objective to minimise the threat of HPAI to humans whilst simultaneously promoting poultry production in the country [15]. Through evaluating the extent to which intersectoral partnerships have been maintained since completion of the project in 2009, this case study examines where One Health may be headed now that the H5N1crisis is over.

#### Institutional outcomes from avian influenza control in Nigeria

The National Technical Committee on Avian Influenza (NTCAI) was established to support interministerial collaboration through joint workshops, training activities, and establishment of desk officers at the state and local government levels. Respondents commented that working in this One Health space was beneficial at the time, with government officials “*opening their eyes*” as to what each sector could offer to the overall fight against H5N1. Others felt that whilst at the Federal level the approach was working well, functionality of the technical committees at the state level varied, appearing weak in some states and making good progress in others. In general however it was felt that the HPAI outbreak of 2006 energized communication between the Ministries of Health and Agriculture, which had been somewhat lacking in recent years; *“This HPAI, it brought us close together and strengthened the bond” (Key Informant 2, Nigeria)*.

#### The future of One Health in Nigeria

Although One Health in Nigeria currently shows “*very good possibilities*” *(Key Informant 3, Nigeria)* it appears far from institutionalised, particularly at the local government level. This is significant given the logistical challenges of human and animal health service delivery in the country's vast rural areas, particularly for the main livestock holding states of the north. Unless One Health policy is agreed and facilitated across all tiers of government, the benefits will be lost where they could be most significant; for example, in the remote rural populations across Africa thought to harbour considerable zoonotic disease burdens [5],[16]–[17]. Furthermore, empirical evidence suggests that whilst the approach is clear and widely accepted within the Nigerian veterinary sector, the medical sector appears yet to be convinced. Many hope the awareness and mutual professional respect generated by Nigeria's H5N1 outbreak and ongoing programmes including the national Field Epidemiology and Laboratory Training Programme (FELTP) mean human health actors “*have no choice now*” but to get on board; “*Previously the medics (sic) were not able to work with anybody…‥this time around, with the One World One Health thing, people have to learn to work together and achieve common goals*” *(Key Informant 4, Nigeria)*.

### Case Study Three: Scale-Out of Pilot Research Projects to Country-Wide Elimination - Rabies in Tanzania

Rabies is widespread in Africa, contributing to an estimated 23 000 deaths per year despite the existence of an effective toolbox for control [18],[19]. Many officials do not prioritise rabies, doubting the feasibility of its elimination through mass dog vaccination [19]. A major question in the recent flurry of One Health activity is how localised academic and scientific projects funded by international donors can move into wider policy spheres in the African context; the ongoing experience of rabies research in Tanzania is illustrative of this process.

#### From the Serengeti to the Selous

Rabies research has been conducted in and around the Serengeti ecosystem since the 1990's, driven by initial concerns regarding rabies outbreaks in the endangered wild dog populations. After confirming that a susceptible domestic dog population is the main driver for outbreaks in wildlife, research programmes turned their attention to domestic canine vaccination, promoting the added benefits to human health and conservation. A series of campaigns over the next decade demonstrated the willingness of dog owners to vaccinate against rabies, even in remote agro-pastoralist communities, and found that coverage of at least 70 percent of the susceptible population was enough to reduce rabies incidence by 90 percent. This corresponded with reductions in the demand for post-exposure prophylactic treatment (PEP) in humans, indicating that cost sharing between human and animal health sectors should occur [20].

Having successfully reduced rabies incidence over more than a decade, researchers were eager to incorporate findings into national policy. This opportunity arose in 2008, through funding from the Bill and Melinda Gates Foundation (BMGF) for a rabies elimination demonstration project in South Africa, the Philippines and Tanzania. The Tanzanian project involved five annual dog vaccination campaigns over 23 districts, covering close to 400 000 dogs and six million people. Unlike previous research projects, this large-scale intervention required involvement and coordination of several key ministries and the Prime Minister's Office. As one researcher commented: *‘As we prepared the project outline together with different ministry people, they were a bit reluctant that rabies was even an issue…it was really the first time we communicated with policymakers” (Key Informant 1, Tanzania)*. National budgets were mobilised alongside the $4 million USD BMGF budget, and the Serengeti researchers hoped to generate sufficient state ownership of the process to ensure canine vaccination could be scaled up to other parts of the country and, if possible, the region; *“We had workshops with ministries and it gave us the opportunity to present our results and people were really surprised, it was totally new for them and they started to express interest (Key Informant 2, Tanzania)*.

#### The challenges of institutionalising country wide elimination

Key informants emphasised that moving from localised research projects managed by academic institutes to a large-scale elimination project embedded within national frameworks and budgets presented many logistical challenges. *“The project really had problems with the number of staff…there was not enough people on the ground to do the vaccination even though according to policy [livestock field officers] should be in every ward; a lot of money was used to pay for per diems (Key Informant 3, Tanzania).”* Besides human resources, infrastructural issues including village access and cold chain maintenance of the vaccine presented additional problems. According to one key informant, some districts interpreted the need for 70 percent coverage as the need to only target 70 percent of villages. In another area a District Veterinary Officer claimed that coverage was well below the required 70 percent. *“The project here has not been able to reach dogs in remote areas where you actually find most of the dogs. The budget just comes to you and you are helpless since every district gets the same amount, even those with few dogs and a small geography” (Key Informant 4, Tanzania)*. It was apparent that *“while the science is there and we have the tools, things are still moving slowly due to problems with capacity and infrastructure.”(Key Informant 5, Tanzania)*.

The Tanzanian case study demonstrates how moving from targeted research projects to national ownership of One Health programmes in Africa will need to navigate weak delivery systems and the accompanying resource limitations. *“We are still trying to find who the right people are to push for a national plan for rabies…but it is difficult…how do you get access to decision-makers and create lasting national ownership?” (Key Informant 6, Tanzania)*. Research plays an important role in driving One Health forwards, but results require appropriate packaging to ensure uptake, particularly at the higher policy levels. One lesson from this case study is the importance of identifying individual government ‘champions’ to drive the institutionalisation process; *“Really, what you need are dedicated ‘rabies champions’ in Tanzania to push for institutional changes in how the ministries work together, share funds and plan…without that, things will be difficult.” (Key Informant 7, Tanzania)*.

#### Lessons from the three case studies

Overall, this series of three African case studies details some of the first empirical evidence demonstrating both the successes and challenges of operationalising One Health in a developing country context through the eyes of national decision makers. It contributes to the requirement for evidence surrounding the *how* rather than the *why* of One Health; *how* to manage health issues across the various representative sectors, particularly in low resource settings where a multitude of human and animal health priorities compete for attention within weak health delivery systems.

The Ugandan case study demonstrates how permanent One Health structures for zoonoses control, whilst desirable as a politically endorsed “glue” to hold everything together, requires strong political commitment and ongoing financial support in order to weather the inter-ministerial “turf wars” [21] likely to emerge from their establishment. Interestingly, zoonotic sleeping sickness was the “avian flu’ of its time in Uganda; demonstrating how the epidemic potential of a disease will likely act as a strong driver for the natural evolution and ownership of a One Health platform. Following this, evidence from the Nigeria case study suggests that whilst a public health crisis serves to facilitate and encourage intersectoral collaboration at the time, things quickly return to “business as usual” if interventions are largely externally-funded and existing government frameworks are not adjusted to support long term change. Finally, the Tanzanian case study details the challenges of rolling out small scale research projects into nationally funded country wide programmes, demonstrating that despite scientific evidence for action, implementing research results on a large scale requires an understanding of national policy processes, adequate capacity, and appropriate packaging of the evidence. The importance of gaining the support of national “champions” is illustrated as a key requirement, which, along with functional animal and human health delivery systems and appropriate socioeconomic evidence for policy, remains a common denominator for successful zoonotic disease control across much of the continent.

### Conclusion

The critical message emerging from all three case studies is that One Health will not “just happen”. Broad institutional changes - and ownership of these changes across the various ministries, departments and interest groups with a stake in disease control – are required for One Health to become a widespread approach to health policy. Moreover, institutional change and ownership will not drive One Health forwards in the absence of sufficient funding. Where external donors are to be the main source of financial support for One Health operationalisation, the need to balance global health agendas with national ownership of change will become even more crucial.

There is no “blanket approach” to One Health; individual country requirements cannot be underestimated, dismissed or prescribed in a top down manner by the international community. Although One Health promotes intersectoral collaboration through flexibility and “small ‘g’ governance” [22], evidence from these three case studies suggest that achieving this in the absence of a global health emergency, political endorsement and nationally-owned financial commitment is at once both challenging, yet urgently required.
